# Unveiling situation‐specific emotion regulation behaviour among teachers: Insights from a multilevel latent profile analysis

**DOI:** 10.1111/bjep.12765

**Published:** 2025-04-02

**Authors:** Tanja Bross, Anne Christiane Frenzel, Ulrike Elisabeth Nett

**Affiliations:** ^1^ University of Augsburg Augsburg Germany; ^2^ Ludwig‐Maximilians‐Universitaet Muenchen Munich Germany

**Keywords:** diary study, emotion regulation, emotions, multilevel latent profile analyses, teachers

## Abstract

**Background:**

In their daily work life, teachers experience various situations in which they need to regulate their emotions. Possible factors that influence the use of different emotion regulation strategies include the emotions and context experienced. Previous research mainly investigated teachers' emotion regulation at a single strategy level without considering multiple strategies use, emotions, situational aspects and well‐being.

**Aims:**

This study aimed to explore teachers' emotion regulation in their daily work life. Furthermore, we examined their emotions, specific situations and well‐being linked to regulation behaviour.

**Sample:**

In total, 165 teachers (mean age 43.31 years; 83.6% female) participated in a diary study for two weeks.

**Methods:**

We assessed eight emotion regulation strategies, joy and anger, the valence and context of the situation and emotional exhaustion. The data were analysed using multilevel latent profile analyses.

**Results:**

We found different combinations of emotion regulation strategy use at the situational level (Level 1) and the person‐level (Level 2). At the person‐level, only a few teachers (17.4%) were able to flexibly apply different strategies in different situations, while most teachers regulated their emotions predominantly with a specific set of strategies. Experiencing joy, as well as the valence and the context of the situation, mattered for emotion regulation at the situational level. Emotional exhaustion at the person‐level was not linked to profile probability.

**Conclusion:**

The findings highlight the complexity of teachers' emotion regulation, revealing diverse regulation patterns and profiles influenced by emotions and situational factors, while underscoring the limited flexibility in strategy use among most teachers.

## INTRODUCTION

In their daily work life, teachers encounter various situations, from challenging to comforting (Schmidt et al., 2017). Depending on their individual perception and interpretation of these situations, teachers experience different emotions. Previous studies have indicated a close connection between teachers' emotions and their general well‐being (Burić et al., 2017; Keller et al., 2014), as well as with student outcomes (Frenzel et al., 2021). Among many relevant and important emotions, joy and anger have been found to be the most commonly encountered emotions among teachers (Frenzel, 2014). In managing their emotions, teachers employ a range of emotion regulation strategies (Taxer & Gross, 2018). While it is theoretically and empirically recognized that the experience and regulation of emotions are emotion‐ and situation‐specific (Rottweiler et al., 2018, 2023), little is understood about how different patterns of emotion regulation strategies are used by teachers in specific work‐related situations. To gain a better understanding, we conducted a diary study to identify patterns of situation‐specific emotion regulation among teachers concerning two emotions (enjoyment and anger), the valence of the situation (positive vs. negative), the context (teaching vs. non‐teaching situations) and subjective well‐being.

### Teachers' emotions

According to previous research, enjoyment is the most frequently reported positive emotion, while anger is the most commonly reported negative emotion among teachers (Burić & Frenzel, 2019; see Frenzel, 2014, for an overview; Frenzel & Goetz, 2007; Keller et al., 2014). Most studies on teachers' emotions have focused on the emotions experienced during teaching (Frenzel, 2014). Research also indicates that teachers tend to experience positive emotions more frequently than negative ones (Becker et al., 2015; Frenzel & Goetz, 2007; Keller et al., 2014). However, this might suggest a bias, as the teaching profession, possibly due to its high ideals, may discourage the acknowledgement of negative emotions associated with teaching (e.g., Sutton, 2004).

### Teachers' emotion regulation

Emotion regulation in general encompasses ‘the processes by which individuals influence which emotions they have, when they have them, and how they experience and express these emotions’ (Gross, 1998b, p. 275). A vivid and ongoing scientific debate about the classification and function of emotion regulation strategies has brought forward different emotion regulation models (e.g., Gross, 1998b; Koole, 2009; Parkinson & Totterdell, 1999). The most comprehensive and widely adopted model of emotion regulation has been proposed by Gross (Gross, 1998a, 1998b, 2015). This model adopts a temporal arrangement of strategies and describes emotion regulation as a dynamic process, categorizing it into two strategy classes, the antecedent‐focused and the response‐focused class. The antecedent‐focused strategy class comprises the strategy families involving situation selection, situation modification, attentional deployment and cognitive change. The response‐focused strategy class comprises the strategy family involving response modulation. Situation selection strategies involve taking action to bring about a different and more favourable situation (e.g., behavioural avoidance (1)). Situation modification strategies entail altering the situation to change its emotional impact (e.g., taking action to change the situation (2), seeking social support from others (3)). Attentional deployment involves directing attention towards or away from the task or emotional stimuli (e.g., distraction (4), rumination^1^ (5)). Cognitive change refers to altering one's thoughts about the self or the situation to influence one's emotions (e.g., reappraisal (6)). Lastly, response modulation strategies aim to directly adjust the parameters of emotional response (e.g., suppression (7), expression (8)) once an emotion has already arisen.

Building on Gross's model (1998b), Harley et al. (2019) outlined an integrated model of emotion regulation in achievement situations (ERAS model), which links Gross's process model (1998b) with control‐value theory (Pekrun, 2006). This model could also be adapted to teachers' experiences at work, as teachers presumably strive to be successful in their profession. The school context also might serve as an achievement context for teachers (Butler, 2007), and therefore, teachers' emotions could be considered achievement emotions. In the ERAS model (Harley et al., 2019), achievement situations are differentiated according to the features of interaction with others (individual vs. social) and the level of evaluation (low vs. high). An individual situation for teachers might be the completion of administrative tasks, while teaching could be considered a social situation. In addition, most administrative tasks would represent a low‐evaluated situation for teachers, while whether teaching is perceived as a low‐ or high‐evaluated situation might depend on subjective perception and the situation itself. Within these relevant situations, attention is directed towards the performance activity or the performance result and according to a specific time frame, that is, the temporal perspective of the activity or the result (prospective, present and retrospective). The situation is then evaluated, with control‐ and value‐appraisals acting as central mechanisms. These evaluations lead to a discrete achievement emotion being experienced, the emotional reaction. The ERAS model (Harley et al., 2019) further elaborates that within the phases of the emotional experience in a specific situation, different emotion regulation strategies might be applied simultaneously or in a very short sequence. This highlights the importance of not examining individual strategies in isolation or assuming a fixed, sequential order. Instead, it underscores the relevance of the dynamic and flexible nature of emotion regulation. There are no specific models for the emotion regulation of teachers, but Gross's (1998b) and Harley et al.'s (2019) models appear highly applicable for explaining emotion regulation within the specific context of teachers' everyday professional lives.

### Empirical findings on teachers' emotion regulation

To date, there are multiple findings on the effectiveness of distinct strategies proposed within Gross's model (1998b), while many studies predominantly focused on reappraisal and suppression. Previous research hints that reappraisal can be regarded as an adaptive strategy and suppression rather as a maladaptive strategy (e.g., Gross & John, 2003).

For teachers, empirical evidence further suggests that suppression is linked to higher emotional exhaustion (Chang, 2020; Doyle et al., 2024; Yin et al., 2016), stress (Jeon & Ardeleanu, 2020), depersonalization, and inefficiency (Chang, 2020), and lower personal accomplishment (Doyle et al., 2024) and teaching satisfaction (Yin et al., 2016), whereas reappraisal is associated with higher positive affect (Messineo & Tosto, 2022), personal accomplishment (Doyle et al., 2024) and teaching satisfaction (Yin et al., 2016), less stress (Jeon & Ardeleanu, 2020; Messineo & Tosto, 2022), emotional exhaustion (Doyle et al., 2024; Yin et al., 2016), depersonalization, and inefficiency (Chang, 2020) and negative affect (Messineo & Tosto, 2022). A recent meta‐analysis by Wang et al. (2023) found that antecedent‐focused strategies were positively associated with well‐being, whereas response‐focused strategies were negatively associated with well‐being and teaching effectiveness. Moreover, teachers' emotion regulation seems to influence their classroom practices and their students' well‐being (Sutton & Knight, 2006). Teachers' use of suppression is associated with a lower positive outlook and less prosocial behaviour among students (Braun et al., 2020) as well as lower classroom management and supportive climate (Burić & Frenzel, 2020).

### Situation‐specificity of teachers' emotion regulation

The cumulative evidence described above typically rests on a habit‐based, person‐typical inquiry to emotion regulation, asking individuals how they generally manage their emotions. As a result, this research implicitly suggests that individuals use either one of those strategies, or strategy families, with particular frequency, and hence profit or suffer in terms of their well‐being. However, individuals likely employ multiple emotion regulation strategies either simultaneously or in a very close sequence (Ford et al., 2019; Heiy & Cheavens, 2014; Sutton, 2004; Taxer & Gross, 2018). A few studies have investigated habitual tendencies for emotion regulation addressing combinations of multiple strategies in teachers so far: Chang and Taxer (2020), Li et al. (2023) and Yin and Guo (2024) found differential combinations of strategy use. Importantly, none of these three studies considered the situational context. Thus, these studies showed that teachers report using different emotion regulation strategies to different degrees, with some engaging in multiple emotion regulation strategies frequently and others reporting to predominantly use one or the other emotion regulation family. However, these studies do not indicate which strategies are used combined in a given situation.

As such, adaptive emotion regulation might not be about the predominance of one emotion regulation strategy use over another but rather about a flexible and situation‐specific strategy use (e.g., Aldao, 2013; Chang & Taxer, 2020; Fombouchet et al., 2023; Rottweiler et al., 2018, 2023; Troy et al., 2013). A better understanding of emotion regulation can likely be achieved by adopting a person‐centered approach coupled with a situated method of inquiry, which allows for modelling different emotion regulation strategy combinations across different groups of situations. Multilevel latent profile analysis is a promising tool for such an approach to exploring emotion regulation.

One study on adults by Grommisch et al. (2020) investigated the use of nine strategies and revealed nine different combinations of emotion regulation at the behavioural (situational) level and five different combinations at the habitual (person) level, revealing a specific personal tendency to apply these behavioural combinations of emotion regulation strategies. Rottweiler et al. (2023) investigated eight emotion regulation strategies in students' regulation of academic emotions versus general emotions and found five combinations at the behavioural (situational) level and three at the habitual (person) level. In addition, Rottweiler et al. (2023) demonstrated that students' behavioural emotion regulation is situation‐specific, as they found that students employed different regulation strategies in achievement and non‐achievement situations. So far, no studies have examined the behavioural and habitual tendencies in teachers' emotion regulation.

### Emotion‐specificity of teachers' emotion regulation

Gross (1998b) typically speaks very generically of ‘emotion regulation’. However, recent theoretical and empirical evidence also suggests that different emotions might be regulated in distinct ways and that certain emotion regulation strategies might be more effective for one, but less effective for another emotion. Rottweiler et al. (2018) found that university students' suppression could alleviate their anxiety experienced in exam‐related situations, while their use of attentional deployment seemed effective for regulating anxiety only in non‐exam‐related situations. Furthermore, Rottweiler et al. (2023) revealed that emotion regulation profiles are emotion‐specific, meaning that the intensity of specific emotional experiences might elicit specific emotion regulation behaviour.

Smrtnik Vitulić and Prosen (2022) found that teachers used situation modification, experiential response modulation (e.g., switching off the emotion) and physical activation (e.g., going for a walk to decrease emotion) more frequently to regulate their anger than fear, while suppression was used more frequently to regulate fear than anger. Meihami and Esmaili (2024) used a qualitative approach to show that teachers used different strategies to regulate their negative emotions like anxiety, anger, fear, sadness and confusion.

Joy and anger are two activity emotions, which are emotions related to current situations (Pekrun, 2006), both are activating and cover positive and negative valence (Pekrun et al., 2002), and they are further found empirically to be the most prominent emotions in teachers (Burić & Frenzel, 2019; see Frenzel, 2014, for an overview; Frenzel & Goetz, 2007; Keller et al., 2014). Thus, we considered it worthwhile to start investigating teachers' emotion regulation behaviour towards specific emotions by focusing on these two emotions.

In summary, previous empirical findings indicate that emotion regulation is highly complex, with varying emotion regulation patterns and profiles that are both situation‐ and emotion‐specific. This complexity is also presumed to apply to teachers.

### The present study

The aim of the present study was to investigate teachers' emotion regulation in their daily work life. We considered that teachers might use different patterns of emotion regulation strategies and that their emotion regulation might depend on their emotional experiences and the specific situation–for example, whether the regulation patterns differ when teaching versus engaging in other activities. Given the limited prior research on teachers' emotion regulation that considers both situational and personal aspects, the current approach was largely exploratory in nature. The following research questions were addressed:

1. What patterns and profiles of emotion regulation can be identified among teachers at the situational and the person‐level? Hereby, we are referring to patterns as combinations of strategies employed towards one specific challenge (situational level), and to profiles, meaning individual tendencies to regulate in a specific way (person‐level). We expect these patterns to be characterized by a variety of emotion regulation strategies rather than simply low and high levels of regulation. We anticipate that teachers differ interindividually in their use of these situational level patterns.
2. How are the different situational level patterns tailored to specific emotions (joy and anger), the valence of the situation (positive or negative) and the context (teaching or non‐teaching)?
3. How are the different person‐level profiles linked to teachers' emotional exhaustion?

For research questions two and three, we have no specific assumptions about the differences. Therefore, we will conduct exploratory tests with undirected hypotheses.

## METHOD

### Sample and procedure

A total of 165 teachers from German elementary (*N* = 86, 52.2%) and vocational^2^ track secondary schools (*N* = 78, 47.3%, one teacher did not indicate the school) were involved in a diary study that spanned two weeks of assessment. Each day, teachers first indicated if the present day had been a workday. The participants had a mean age of 43.31 years (*SD* = 11.12; min = 22, max = 66 years), with 83.6% identifying as female (*n* = 138), and the rest as male. On average, teachers reported 16.02 years of teaching experience (*SD* = 10.32; min = 1, max = 43 years). Out of 2,303 daily questionnaires distributed, 1,604 (69.6%) were answered. Among these questionnaires, 1,159 (72.4%) were completed on workdays and were thus considered for analysis. On average, each participant filled out 7.02 questionnaires related to workdays.

The study received approval from the local school district. Informed consent was obtained from all participants. Daily questionnaires were emailed, and participants received monetary compensation and individual feedback upon completion.

Participants completed a questionnaire on demographics both on the initial day and the concluding day. During the intervening fourteen days, a brief questionnaire was distributed each evening for completion between 6 p.m. and midnight on that particular day.

### Measures

#### Situations

Each day, the teachers were asked to describe up to four emotionally intense work situations in an open format (Schmidt et al., 2017). In the first step, these situations were coded by two raters (Gwet's AC1 = .84) (Gwet, 2008) using a coding scheme adapted from Schmidt et al. (2017), categorizing them into six different categories (teaching, interaction with colleagues, organization, preparation, interaction with parents, and other). In the second step, the situations were dummy coded (Gwet's AC1 = .89) (Gwet, 2008) as either teaching‐related (e.g., ‘relaxed atmosphere in own classroom’) or non‐teaching‐related, encompassing all other categories (e.g., friendly comment from a student's mother). A total of 2,209 situations were assessed, with *n* = 1,018 classified as teaching situations and *n* = 1,126 as non‐teaching situations (*n* = 65 situations were missing).

Teachers were then instructed to select one emotionally positive and one emotionally negative situation from the up to four situations they had reported, with the following instruction: ‘Please choose the situation that you remember as particularly positive/negative’. These selections served as indicators of the valence of the situation, which was dummy coded as 0 for negative and 1 for positive situations. Emotions and emotion regulation strategies were subsequently assessed for both situations.

#### Emotions

In relation to each situation, joy and anger were assessed using single items (Goetz et al., 2013; Rottweiler et al., 2018) with the item stem ‘With regard to this positive/negative situation, I felt … [joy, anger]’ on a five‐point Likert scale (0: do not agree to 4: agree).

#### Emotion regulation strategies

For each situation, eight emotion regulation strategies were assessed, using single items on a five‐point Likert scale (0: do not agree to 4: agree) adapted from Carver et al. (1989), Garnefski and Kraaij (2007), Gross and John (2003) and Rottweiler et al. (2018). The items represent the five strategy families of Gross's (1998b) process model, namely (1) *situation selection*, (2) *situation modification*, (3) *attentional deployment*, (4) *cognitive change* and (5) *response modulation*. To operationalize situation selection, we assessed avoidance (‘In this situation, I did something to distract myself’). Within the situation modification family, we differentiated between the two sub‐strategies of taking action (‘In this situation, I took action to improve the situation’) and social support (‘I shared this situation/my feelings about this situation with others’). Within the family of attentional deployment, we differentiated between the two sub‐strategies of distraction (‘In this situation, I distracted myself in my thoughts’) and rumination (‘I couldn't get this situation out of my mind all day today’). To operationalize cognitive change, we assessed reappraisal (‘In this situation, I made myself aware of the positive aspects of the situation’). Within the family of response modulation, we differentiated between the two sub‐strategies of suppression (‘In this situation, I kept my feelings to myself’) and expression (‘In this situation, I let my feelings run free’).

#### Emotional exhaustion

The last section of the diary was devoted to the assessment of emotional exhaustion, which was measured using four items (Maslach et al., 1996) on a four‐point Likert scale (0: do not agree to 3: agree) (*ICC(1)* = .517). A sample item was ‘I feel emotionally drained from my work’.

### Analyses

We ran multilevel latent profile analyses (ML‐LPA) with the software Latent GOLD Version 6.0 (Vermunt & Magidson, 2021) to identify the emotion regulation patterns at Level 1 (*n* = 2,136) and the profiles at Level 2 (*n* = 165). The strategies were not grouped together into five strategy families in the analyses but were included as individual strategies. We followed the three‐step procedure proposed by Lukočienė et al. (2010). To avoid local maxima, we used 1,000 random sets of starting values (Grommisch et al., 2020; Hipp & Bauer, 2006; Rottweiler et al., 2023) and 500 iterations. To determine the best model and assess the quality of profile separation, we considered the log‐likelihood (LL), Bayesian information criterion (BIC; Schwarz, 1978), the Akaike information criterion 3 (AIC3; Bozdogan, 1987), the Vuong–Lo–Mendell–Rubin test (VLMR; Lo et al., 2001; Vuong, 1989), entropy R‐squared, the classification error and the size of the smallest pattern/profile. We also evaluated the interpretability of the patterns and profiles. After identifying the final ML‐LPA model, we included covariates in the analysis using the step 3 modelling procedure implemented in Latent Gold with proportional classification and maximum‐likelihood estimation. To answer research question 2, we included the emotions' intensity (joy and anger, five‐point Likert scale), valence (positive vs. negative), context (teaching vs. non‐teaching), emotion × valence, emotion × context, context × valence and emotion × emotion as covariates at Level 1.

Emotions were person‐mean centered by subtracting the person mean from the time‐varying variable, that is, the person‐specific situational assessment. Context and valence were dummy coded. Emotional exhaustion was grand‐mean centered at Level 2. Missing data were treated by using the full information maximum‐likelihood (FIML) estimator.

## RESULTS

The descriptive statistics for all variables are presented in Table 1. Correlations were tendentially higher at the between‐level than at the within‐level. Moreover, some strategies exhibited similar correlations at both levels, while others differed. For example, at the between‐level, avoidance and distraction were highly correlated (*r* = .92, *p* < .05), implying that individuals who reported using avoidance a lot were also those who frequently reported using distraction as a strategy. Additionally, the between‐level correlation between avoidance and reappraisal was *r* = .44 (*p* < .05), indicating that individuals who frequently used avoidance also engaged in reappraisal more often. At the within‐level, avoidance and distraction were also positively correlated (*r* = .57; *p* < .05), implying that these two strategies were reported simultaneously. However, the within‐level correlation between avoidance and reappraisal was *r* = −.26 (*p* < .05), indicating that when a person reported using avoidance in a given situation, they were less likely to use reappraisal in the same situation.

**TABLE 1:** Descriptive statistics.

		*N*	*M*	*SD*	*ICC(1)*	*ICC(2)*	1	2	3	4	5	6	7	8	9	10	11	12^a^	13^b^
1	Avoidance	2191	0.51	0.97	.216	.787		.15	.**22**	.**92**	.**55**	.**44**	.08	.**40**	.81	.11	**−.36**		**−.38**
2	Taking Action	2187	2.20	1.49	.172	.735	.**06**		.**49**	.02	.16	.**82**	−.15	.**25**	.80	−.37	−.14		−.02
3	Social Support	2194	2.07	1.54	.226	.796	**−.06**	.**16**		.19	.**35**	.**48**	**−.63**	.**70**	.59	.**70**	−.11		−.02
4	Distraction	2192	0.62	1.10	.147	.698	.**57**	.02	**−.14**		.**41**	.**36**	.12	.**36**	.75	−.03	−.23		**−.30**
5	Rumination	2203	1.40	1.38	.230	.800	.**08**	.02	.**31**	.02		.**40**	−.10	.**37**	−.01	.**79**	−.18		**−.40**
6	Reappraisal	2200	2.02	1.60	.105	.611	**−.23**	−.05	.**10**	**−.15**	−.01		−.08	.**37**	.08	.60	−.03		.21
7	Suppression	2206	1.86	1.49	.139	.683	.**18**	.00	**−.39**	.**32**	**−.14**	**−.13**		**−.67**	.09	**−.97**	−.09		−.04
8	Expression	2194	1.45	1.38	.264	.828	**−.13**	.**09**	.**38**	**−.21**	.**20**	.**27**	**−.49**		.57	.**72**	−.06		.02
9	Joy	2204	1.87	1.70	.000	.000	**−.32**	**−.17**	.**09**	**−.30**	−.04	.**64**	**−.26**	.**33**		.87	−.33		.91
10	Anger	2199	1.37	1.59	.011	.135	.**28**	.**20**	.**07**	.**27**	.**15**	**−.54**	.**15**	**−.15**	**−.73**		.58		**−.96**
11	Context	2144	0.48	0.50	.075	.521	**−.11**	.**08**	.05	**−.13**	−.04	.04	**−.18**	.**08**	.**14**	−.06			.15
12	Valence^a^	2209	0.51	0.50	.000	.000	**−.32**	**−.17**	.**07**	**−.32**	−.06	.**57**	**−.26**	.**29**	.**82**	**−.73**	.**09**		
13	Emotional Exhaustion^b^	1153	1.84	0.77	.473	.863													

*Note*: Theoretical range for emotions and regulation strategies: 0–4, theoretical range for context and valence: 0–1, context: 0 = non‐teaching, 1 = teaching, valence: 0 = negative, 1 = positive, theoretical range for emotional exhaustion: 0–3. Pearson's Correlations with *N* = 165 (Level 2), upper triangular: person‐level (Level 2), lower triangle: situation‐level (Level 1), statistically significant coefficients at *α* < .05 are boldface.

Abbreviations: *M*, mean; *N*, sample size at lower level; *SD*, standard deviation; *ICC(1)*, proportion of variance between individuals; *ICC(2)*, reliability of aggregated variables.

^a^ Valence has no variance at Level 2; hence, correlations were only calculated at Level 1.

^b^ Emotional exhaustion was assessed as a day‐specific variable; hence, correlations were only calculated at Level 2.

### Pattern and profile identification

The model fit statistics for step one (Level 1, ignoring the multilevel structure) are shown in Table 2. As model complexity increased, LL increased, while BIC and AIC3 decreased, and the VLMR test was significant for all models. However, from pattern 6 onwards, the classification errors increased considerably (values above .50), entropies fell below .99, and the size of the smallest profiles dropped below 7%. Based on these indices and considerations of parsimony, the five‐pattern solution was selected. Next, we considered the multilevel structure (step 2). We fixed the number of Level 1 patterns at five and extracted models with up to eight Level 2 profiles (Table 3). As model complexity increased, LL increased, while BIC and AIC3 decreased until the four‐profile solution, where the indices reached their lowest values. From Model 4 to Model 5, however, the VLMR test was no longer significant. At Profile 6, the VLMR test became significant again (*p* < .05), but LL was lower and BIC and AIC3 were higher than in the four‐profile solution. At Profile 8, LL reached its highest value, BIC and AIC3 dropped again, and the VLMR test was significant once more. However, the eight‐profile solution had a higher classification error at Level 2 than the four‐profile solution (0.2451 vs. 0.1555), lower entropy at Level 2 than the four‐profile solution (0.6796 vs. 0.7123) and the size of the smallest profile at Level 2 accounted for only 8.03%, compared to the size of the smallest Level 2 profile for the four‐profile solution with 22.35%. Therefore, and considering parsimony, the four‐profile solution was selected.

**TABLE 2:** Step 1: Model fit statistics for LPA models with different numbers of level 1 patterns.

Nr of patterns (L1)	LL	BIC (LL)	Size of drop in BIC	AIC3 (LL)	Npar	VLMR	*p*	Classification error (L1)	Entropy *R* ^2^ (L1)	Size of smallest P (L1)
1	−30226.21	60575.63		60500.42	16			0.0000	1.0000	1.0000
2	−19158.62	38571.35	22004.27	38416.24	33	22135.18	.0000	0.0012	0.9961	0.3599
3	−17070.46	34525.93	4045.43	34290.91	50	4176.33	.0000	0.0018	0.9947	0.1585
4	−15746.81	32009.55	2516.38	31694.63	67	2647.29	.0000	0.0034	0.9903	0.0645
**5**	**−14611.14**	**29869.10**	**2140.44**	**29474.28**	**84**	**2271.35**	.**0000**	**0.0050**	**0.9895**	**0.1052**
6	−13935.79	28649.30	1219.80	28174.57	101	1350.70	.0000	0.0231	0.9619	0.0530
7	−13065.51	27039.66	1609.64	26485.03	118	1740.55	.0000	0.0083	0.9829	0.0470
8	−12195.44	25430.43	1609.23	24795.89	135	1740.14	.0000	0.0098	0.9837	0.0616

*Note*: Bold numbers show the best pattern solution.

Abbreviations: L1, Level 1; LL, log‐likelihood; Npar, number of parameters.

**TABLE 3:** Step 2: Model fit statistics for ML‐LPA models with different numbers of level 2 profiles (and five level 1 patterns).

Nr of profiles (L2)	LL	BIC (LL)	Size of drop in BIC	AIC3 (LL)	Npar	VLMR	*p*	Classification error (L1)	Entropy *R* ^2^ (L1)	Classification error (L2)	Entropy *R* ^2^ (L2)	Size of smallest P (L2)
1	−14708.14	30063.11		29668.28	84			0.0049	0.9890	0.0000	1.0000	1.0000
2	−14461.52	29608.37	454.74	29190.04	89	493.24	.0000	0.0057	0.9886	0.0830	0.7411	0.4591
3	−14186.21	29096.26	512.11	28654.43	94	550.61	.0000	0.0059	0.9886	0.1286	0.7231	0.2026
**4**	**−14153.32**	**29068.97**	**27.29**	**28603.64**	**99**	**65.79**	.**0003**	**0.0057**	**0.9886**	**0.1555**	**0.7123**	**0.2235**
5	−14309.14	29419.12	−350.15	28930.29	104	−311.64	1.0000	0.0043	0.9903	0.1801	0.7116	0.1292
6	−14295.71	29430.76	−11.64	28918.42	109	26.86	.0384	0.0044	0.9904	0.2011	0.7035	0.1318
7	−14355.09	29588.01	−157.25	29052.17	114	−118.75	1.0000	0.0053	0.9890	0.2115	0.7003	0.0775
8	−14109.66	29135.65	452.36	28576.31	119	490.86	.0000	0.0061	0.9885	0.2451	0.6796	0.0803

*Note*: Bold numbers show the best profile solution.

Abbreviations: L1, Level 1; L2, Level 2; LL, log‐likelihood; Npar, number of parameters.

In the third step, we reanalysed the Level 1 patterns while fixing the Level 2 profiles at four (Table 4). Again, as model complexity increased, LL increased, while BIC and AIC3 decreased. The VLMR test was significant for all models. However, from the six‐pattern solution at Level 1 onward, the smallest patterns accounted for less than 7%. Considering all indices at both levels, the five‐pattern solution provided the best overall fit. Consequently, we selected the five‐pattern solution (Level 1) and the four‐profile solution (Level 2) as the final model.

**TABLE 4:** Step 3: Model fit statistics for ML‐LPA models with different numbers of level 1 patterns (and four level 2 profiles).

Nr of patterns (L1)	LL	BIC (LL)	Size of drop in BIC	AIC3 (LL)	Npar	VLMR	*p*	Classification error (L1)	Entropy *R* ^2^ (L1)	Size of smallest P (L1)	Classification error (L2)	Entropy *R* ^2^ (L2)
1	−30226.21	60598.73		60509.42	19			0.0000	1.0000	1.0000	0.7500	0.0000
2	−19015.88	38332.07	22266.65	38148.76	39	22420.66	.0000	0.0011	0.9964	0.3686	0.2417	0.5563
3	−16957.95	34370.23	3961.85	34092.91	59	4115.86	.0000	0.0013	0.9962	0.1131	0.1636	0.6775
4	−15506.01	31620.34	2749.89	31249.01	79	2903.89	.0000	0.0050	0.9879	0.1116	0.1897	0.6552
**5**	**−14329.74**	**29421.82**	**2198.52**	**28956.49**	**99**	**2352.53**	.**0000**	**0.0045**	**0.9900**	**0.1047**	**0.1434**	**0.7296**
6	−13455.20	27826.74	1595.08	27267.40	119	1749.09	.0000	0.0049	0.9891	0.0651	0.1578	0.7222
7	−12919.39	26909.11	917.62	26255.77	139	1071.63	.0000	0.0090	0.9850	0.0616	0.1453	0.7358
8	−11958.12	25140.60	1768.52	24393.25	159	1922.52	.0000	0.0160	0.9771	0.0575	0.1375	0.7480

*Note*: Bold numbers show the best solution.

Abbreviations: L1, Level 1; L2, Level 2; LL, log‐likelihood; Npar, number of parameters.

### Situational patterns (Level 1)

**FIGURE 1:**
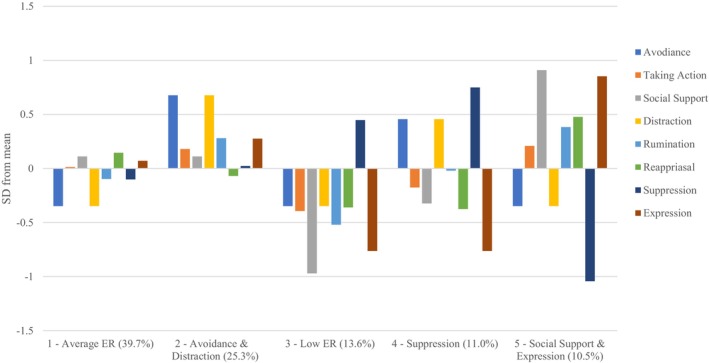
ER patterns at the situational level. Final model with five patterns at the situational level and four profiles at the person‐level. Bars represent the standardized deviation from the sample mean of the specific ER strategy; percentages represent pattern sizes.

Figure 1 shows the different patterns at the situational level (*N* = 2,209). For clarity, we calculated the standardized deviation from the overall mean for each strategy within each pattern. The strategies in the situational patterns thus represent relative deviations from the mean of the strategy. Strategies were considered dominant in the respective pattern if their means deviated by at least 0.5 standard deviations.

Pattern 1 was characterized by taking action (2), social support (3), reappraisal (6) and expression (8) being used only slightly above the mean level, while avoidance (1), distraction (4), rumination (5) and suppression (7) were used only slightly below the mean level. We labelled this pattern ‘Average ER’, which comprised 39.7% (*n* = 877) of situations. In pattern 2, avoidance (1) and distraction (4) were used notably above the mean level, while other strategies did not deviate noticeably. We labelled this pattern ‘Avoidance and Distraction’ (25.3%, *n* = 558) because the pattern focused on avoidance‐oriented regulation (e.g., behavioural and cognitive). In pattern 3, all ER strategies except suppression (7) were used below the mean level. We named this pattern ‘Low ER’, which comprised 13.6% (*n* = 300) of all situations across individuals. Pattern 4 was characterized by above‐average use of suppression (7), while the other strategies were used below the mean level. Accordingly, we labelled it ‘Suppression’ (11.0%, *n* = 243). In pattern 5, social support (3) and expression (8) were used notably above the mean level, while suppression (7), an avoidance‐based strategy, was used below the mean level. We labelled this pattern ‘Social Support and Expression’ (10.5%, *n* = 231).

### Person‐level profiles (Level 2)

**FIGURE 2:**
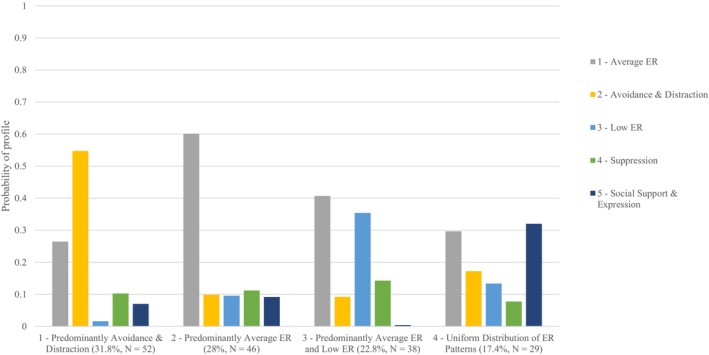
ER profiles at the person‐level. Final model with five patterns at the situational level and four profiles at the person‐level. Percentages represent the profile size with the number of persons.

At the person‐level (*N* = 165), multilevel profile analysis identified a four‐profile solution as the best solution (Table 4). All four person‐level profiles were represented with similar frequencies (Figure 2). The first profile was characterized by the predominant use of the pattern ‘Avoidance and Distraction’ (31.8%, *n* = 52). The second profile most frequently resorted to ‘Average ER’ (28.0%, *n* = 46). The third profile was characterized by the predominant use of the patterns ‘Average ER’ and ‘Low ER’ (22.8%, *n* = 38). The fourth profile was characterized by a uniform distribution of all different ER patterns (17.4%, *n* = 29). It is noteworthy that the entropy at the person‐level was only .7296.

### Inclusion of covariates in the situational patterns (Level 1)

The following analysis compared each pattern against the reference standard of pattern 1 ‘Average ER’. The model with the included covariates joy, anger, valence and context of the situation as well as their two‐way interactions is detailed in Table 5; the pairwise comparisons between all patterns, which were corrected for multiple testing using the Benjamini and Hochberg (1995) procedure, can be found in Table 6.

**TABLE 5:** Pattern probability at level 1 predicted by covariates joy, anger, valence and context.

	Pattern 2 Avoidance & Distraction	*SE*	Pattern 3 Low ER	*SE*	Pattern 4 Suppression	*SE*	Pattern 5 Social Support & Expression	*SE*	Wald	*p*
	*B*		*B*		*B*		*B*			
Intercept	.874	.234	−.362	.232	.452	.245	−2.618	.670	46.192	<.001
Joy	.404	.122	.095	.136	.461	.144	.128	.450	16.207	.003
Anger	−.133	.118	−.356	.166	−.340	.146	.476	.398	10.106	.039
Valence	−1.224	.257	−.247	.301	−2.620	.384	.567	.911	58.718	<.001
Context	−.815	.262	−.395	.310	−1.647	.353	.231	.685	24.275	<.001
Joy × Valence	−.748	.187	−.523	.176	−1.025	.195	.342	.632	32.904	<.001
Anger × Valence	.457	.190	.313	.275	.222	.325	−.549	.769	6.402	.170
Joy × Context	−.251	.141	−.344	.151	−.698	.226	−.082	.327	11.508	.021
Anger × Context	.134	.135	−.107	.174	−.072	.199	−.043	.268	3.029	.550
Context × Valence	.942	.461	.628	.483	2.123	.663	−.279	1.242	10.917	.028
Joy × Anger	−.073	.062	.045	.089	−.208	.084	−.054	.227	10.023	.040

*Note*: Pattern 1 ‘Average ER’ is the reference pattern. Joy and anger are person‐mean‐centered, valence and context are dummy coded, valence: 0 = negative, 1 = positive, context: 0 = non‐teaching, 1 = teaching; depicted are the respective categories 1 (teaching and positive situation). The intercepts in the first row indicate the probability of using each pattern compared to the first pattern when average joy/anger is perceived in negative valenced non‐teaching situations, meaning all covariates have a value of 0.

Abbreviations: *B*, log odds ratios; *SE*, standard error; Wald, result of Wald test; *p*, *p*‐value.

**TABLE 6:** Paired comparisons between the reference pattern 1 and patterns 2, 3, 4 and 5.

			Wald	*df*	*p*	adj. *p*
**Intercept**						
Pattern	1: Average ER	2: Avoidance + Distraction	13.989	1	<.001	*
Pattern	1: Average ER	3: Low ER	2.439	1	.120	
Pattern	1: Average ER	4: Suppression	3.422	1	.064	
Pattern	1: Average ER	5: Social Support + Expression	15.248	1	<.001	*
**Joy**						
Pattern	1: Average ER	2: Avoidance + Distraction	10.962	1	.001	*
Pattern	1: Average ER	3: Low ER	0.483	1	.490	
Pattern	1: Average ER	4: Suppression	10.218	1	.001	*
Pattern	1: Average ER	5: Social Support + Expression	0.081	1	.780	
**Anger**						
Pattern	1: Average ER	2: Avoidance + Distraction	1.264	1	.260	
Pattern	1: Average ER	3: Low ER	4.595	1	.032	
Pattern	1: Average ER	4: Suppression	5.406	1	.020	
Pattern	1: Average ER	5: Social Support + Expression	1.432	1	.230	
**Valence**						
Pattern	1: Average ER	2: Avoidance + Distraction	22.746	1	<.001	*
Pattern	1: Average ER	3: Low ER	0.673	1	.410	
Pattern	1: Average ER	4: Suppression	46.443	1	<.001	*
Pattern	1: Average ER	5: Social Support + Expression	0.387	1	.530	
**Context**						
Pattern	1: Average ER	2: Avoidance + Distraction	9.642	1	.002	*
Pattern	1: Average ER	3: Low ER	1.623	1	.200	
Pattern	1: Average ER	4: Suppression	21.779	1	<.001	*
Pattern	1: Average ER	5: Social Support + Expression	0.114	1	.740	
**Joy × Valence**						
Pattern	1: Average ER	2: Avoidance + Distraction	15.990	1	<.001	*
Pattern	1: Average ER	3: Low ER	8.787	1	.003	*
Pattern	1: Average ER	4: Suppression	27.593	1	<.001	*
Pattern	1: Average ER	5: Social Support + Expression	0.292	1	.590	
**Anger × Valence**						
Pattern	1: Average ER	2: Avoidance + Distraction	5.821	1	.016	
Pattern	1: Average ER	3: Low ER	1.292	1	.260	
Pattern	1: Average ER	4: Suppression	0.468	1	.490	
Pattern	1: Average ER	5: Social Support + Expression	0.510	1	.480	
**Joy × Context**						
Pattern	1: Average ER	2: Avoidance + Distraction	3.178	1	.075	
Pattern	1: Average ER	3: Low ER	5.232	1	.022	
Pattern	1: Average ER	4: Suppression	9.562	1	.002	*
Pattern	1: Average ER	5: Social Support + Expression	0.063	1	.800	
**Anger × Context**						
Pattern	1: Average ER	2: Avoidance + Distraction	0.987	1	.320	
Pattern	1: Average ER	3: Low ER	0.381	1	.540	
Pattern	1: Average ER	4: Suppression	0.132	1	.720	
Pattern	1: Average ER	5: Social Support + Expression	0.026	1	.870	
**Context × Valence**						
Pattern	1: Average ER	2: Avoidance + Distraction	4.171	1	.041	
Pattern	1: Average ER	3: Low ER	1.688	1	.190	
Pattern	1: Average ER	4: Suppression	10.267	1	.001	*
Pattern	1: Average ER	5: Social Support + Expression	0.050	1	.820	
**Joy × Anger**						
Pattern	1: Average ER	2: Avoidance + Distraction	1.347	1	.250	
Pattern	1: Average ER	3: Low ER	0.249	1	.620	
Pattern	1: Average ER	4: Suppression	6.170	1	.013	
Pattern	1: Average ER	5: Social Support + Expression	0.057	1	.810	

*Note*: A correction for multiple testing following the Benjamini and Hochberg (1995) procedure was applied and is depicted in the column ‘adj. *p*’ The asterisks indicate significance following this correction with *p < .05.

Abbreviations: Wald, result of Wald test; *df*, degrees of freedom; *p*, p‐value; adj. *p*, adjusted p‐value.

The intercepts in the first row (Table 5) indicate the probability of each pattern's utilization compared to the first pattern ‘Average ER’ if all covariates have a value of zero–that is, when experiencing average joy and anger in negative, non‐teaching situations. The significant Wald test indicates that the patterns differ significantly from each other in their probability. A positive sign indicates an increase in the probability of this pattern compared to pattern 1, a negative sign indicates a decrease in the probability of this pattern compared to pattern 1. Pairwise comparisons (see Table 6) showed that when all covariates have a value of zero, the probability of pattern 2 ‘Avoidance and Distraction’ increases in comparison to pattern 1 ‘Average ER’ (log OR = .874, Wald test *χ* ^2^(1) = 13.989, *p* < .001), and the probability for pattern 5 ‘Social Support and Expression’ decreases in comparison to pattern 1 ‘Average ER’ (log OR = − 2.618, Wald test *χ* ^2^(1) = 15.248, *p* < .001).

We will report the results for each covariate first, followed by a description of the two‐way interaction effects. In situations where teachers experience *joy* more intensively than average, while all other covariates and interactions are set to zero (meaning average anger in negative, non‐teaching situations), the pattern probabilities compared to pattern 1 ‘Average ER’ differed significantly (Wald test *χ* ^2^(44) = 16.207, *p* = .003). Pairwise comparisons with corrected *p*‐values showed that the probability of pattern 2 ‘Avoidance and Distraction’ (log OR = .404, Wald test *χ* ^2^(1) = 10.962, *p* = .001) and the probability of pattern 4 ‘Suppression’ (log OR = .461, Wald test *χ* ^2^(1) = 10.218, *p* = .001) further increased compared to pattern 1 ‘Average ER’ (see Tables 5 and 6).

For situations where teachers experience *anger* more intensively than average, while all other covariates and interactions are set to zero, and after controlling for multiple testing, none of the pairwise comparisons were significant (see Table 6).

In *positive* (as opposed to negative) *situations*, with average joy, anger and a non‐teaching context, the pattern probabilities compared to pattern 1 ‘Average ER’ showed a significant difference (Wald test *χ* ^2^(44) = 58.718, *p* < .001; see Table 5). The probability of pattern 2 ‘Avoidance and Distraction’ (log OR = −1.224, Wald test *χ* ^2^(1) = 22.746, *p* < .001; see Tables 5 and 6) and 4 ‘Suppression’ (log OR = −2.620, Wald test *χ* ^2^(1) = 46.443, *p* < .001; see Tables 5 and 6) decreased compared to pattern 1 ‘Average ER’, making these two patterns less probable than pattern 1 ‘Average ER’.

In situations where teachers were *teaching*, as opposed to conducting non‐teaching tasks such as administration or interaction with colleagues, with average joy, anger and negative valence, the pattern probabilities differed significantly from pattern 1 ‘Average ER’ (Wald test *χ* ^2^(44) = 24.275, *p* < .001, see Table 5). The pairwise comparison showed that the probabilities of pattern 2, ‘Avoidance and Distraction’, and pattern 4, ‘Suppression’, decreased compared to pattern 1, ‘Average ER’, with pattern 2, ‘Avoidance and Distraction’, still being more likely than pattern 1, ‘Average ER’ (log OR = −.815, Wald test *χ* ^2^(1) = 9.642, *p* = .002; see Tables 5 and 6), while pattern 4, ‘Suppression’, became less likely than pattern 1, ‘Average ER’ (log OR = −1.647, Wald test *χ* ^2^(1) = 21.779, *p* < .001; see Tables 5 and 6).

The two‐way interactions all had a significant impact on the probability of using specific emotion regulation patterns, except for anger × valence and anger × context.

For experiencing *joy* in *positive situations*, with average anger in non‐teaching situations, the Wald test revealed significant differences (*χ* ^2^(44) = 32.904, *p* < .001; see Table 5). More specifically, pairwise comparisons demonstrated a decrease in the probability of pattern 2, ‘Avoidance and Distraction’ (log OR = −.748, Wald test *χ* ^2^(1) = 15.990, *p* < .001; see Tables 5 and 6), pattern 3, ‘Low ER’ (log OR = −.523, Wald test *χ* ^2^(1) = 8.787, *p* = .003; see Tables 5 and 6), and pattern 4, ‘Suppression’ (log OR = −1.025, Wald test *χ* ^2^(1) = 27.593, *p* < .001; see Tables 5 and 6), compared to pattern 1, ‘Average ER’.

When experiencing *joy* in a *teaching situation*, with average anger and negative valence, the Wald test showed significant differences in pattern probability compared to pattern 1, ‘Average ER’ (*χ* ^2^(44) = 11.508, *p* = .021; see Table 5). Pairwise comparisons revealed that the probability of pattern 4, ‘Suppression’, decreased compared to the probability of pattern 1, ‘Average ER’ (log OR = −.698, Wald test *χ* ^2^(1) = 9.562, *p* = .002; see Tables 5 and 6).

The Wald test identified significant differences (*χ* ^2^(44) = 10.917, *p* = .028) for experiencing *positive teaching situations* characterized by average joy and anger (see Table 5). The probability of pattern 4, ‘Suppression’, was further increased compared to pattern 1, ‘Average ER’ (log OR = 2.123, Wald test *χ* ^2^(1) = 10.267, *p =* .001; see Tables 5 and 6).

For experiencing *joy* and *anger* more intensely in negative, non‐teaching situations, the pairwise comparisons, adjusted for multiple testing, showed no significant differences in pattern probabilities. Overall, the covariate analyses indicate that the use of distinct regulation patterns is emotion‐ and situation‐specific.

### Inclusion of covariates in the person profiles (Level 2)

Results for the inclusion of emotional exhaustion as a covariate on the profile probability at Level 2 are displayed in Table 7. Profiles did not differ in their levels of reported emotional exhaustion (Wald *χ* ^2^(6) = 7.399, *p* = .060).

**TABLE 7:** Profile probability at level 2 predicted by emotional exhaustion.

	Profile 1 Predominantly Avoidance & Distraction	*SE*	Profile 3 Predominantly Average ER and Low ER	*SE*	Profile 4 Uniform distribution of ER patterns		Wald	*p*
	*B*		*B*		*B*	*SE*		
Intercept	.047	.249	−.204	.271	−.527	.299	4.398	.220
Emotional Exhaustion	−.825	.427	−.198	.439	.511	.484	7.399	.060

*Note*: Profile 2 ‘Predominantly Average ER’ is the reference profile. Emotional exhaustion is grand‐mean‐centered. The intercepts in the first row indicate the probability of using each profile compared to the profile 2 ‘Predominantly Average ER’ when average emotional exhaustion is perceived, meaning emotional exhaustion has a value of 0.

Abbreviations: *B*, log odds ratios; *SE*, standard error; Wald, result of Wald test; *p*, p‐value.

## DISCUSSION

In the present study, we sought to gain insights into how teachers regulate their emotions over a two‐week period, hence using a situated inquiry approach to emotion regulation. Thereby, we went beyond previous research that primarily focused on individual differences in the habitual use of single emotion regulation strategies by teachers (Chang, 2020; Doyle et al., 2024; Jeon & Ardeleanu, 2020; Messineo & Tosto, 2022; Wang et al., 2023; Yin et al., 2016) or combinations thereof (Chang & Taxer, 2020; Li et al., 2023; Yin & Guo, 2024). This approach allowed us to advance research on teachers' emotion regulation by additionally exploring intra‐individual variations in strategy use across different situations.

### Situational patterns

At the situational level, we found five different patterns of emotion regulation strategies. The fact that different patterns of strategy combinations emerged at the situational level is in line with theoretical assumptions (Gross, 1998b; Harley et al., 2019) and previous findings (Ford et al., 2019; Heiy & Cheavens, 2014; Sutton, 2004; Taxer & Gross, 2018) that emphasize the importance of dynamic and flexible emotion regulation. Notably, in four out of the five patterns (patterns 1, 2, 4 and 5), strategies from different emotion regulation families (Gross, 1998b) were combined.

Even though the strategies in pattern 1, ‘Average ER’, were only moderately used, strategies such as taking action, seeking social support, reappraisal and expression were applied. These strategies belong to three of the five emotion regulation families (situation modification, cognitive change and reaction modulation) and represent approach‐based strategies (Gross, 1998b). Most situations were assigned to this pattern (39.7%), indicating that teachers utilized multiple strategies to regulate emotions in various situations, though these strategies were used only at around the mean level. Patterns 2, ‘Avoidance & Distraction’, and 4, ‘Suppression’, were characterized by the predominant use of avoidance‐based strategies pertaining to three different emotion regulation families (situation selection, attentional deployment and reaction modulation). These strategies are defined by avoiding the situation either at a behavioural level (avoidance), a cognitive level (distraction) or by suppressing the experienced emotion (suppression). Unlike pattern 2 ‘Avoidance & Distraction’, where other strategies such as taking action, social support, rumination, suppression and expression were used slightly above the mean, pattern 4, ‘Suppression’, exclusively contained three avoidance‐based strategies with intensities above the mean. In contrast to pattern 4, ‘Suppression’, pattern 3, ‘Low ER’, was predominantly characterized by the use of a single strategy: suppression. According to the process model (Gross, 1998b), suppression is considered a response‐focused strategy, typically employed as a ‘last resort’ when the emotion is already being experienced. This finding suggests that in certain situations (11%), teachers may have had no other strategies available or deemed other strategies inappropriate but still felt the need to regulate their emotions, opting to suppress their emotional expression instead. Pattern 5, ‘Social Support & Expression’, was marked by a stronger use of social support and expression, two strategies associated with displaying emotions, either socially or expressively. These strategies belong to the situation modification and response modulation families. At the same time, this pattern was characterized by a lower‐than‐average use of avoidance strategies (avoidance, distraction and suppression), which may indicate that the combined use of social support and expression reduces the likelihood or necessity of using avoidance‐based strategies.

The identification of these patterns provides empirical support for both Gross's process model (Gross, 1998b) and the ERAS model (Harley et al., 2019). Both emphasize that emotion regulation is a process involving not just the application of a single strategy, but rather a bundle of strategies. Thus, when evaluating emotion regulation behaviour, it may be insufficient to focus solely on individual strategies.

Our findings require further replication but as they are already very similar to the five patterns observed by Rottweiler et al. (2023), they reveal intriguing patterns and interplays among strategies. Pattern 1, ‘Average ER’, and 3, ‘Low ER’, were found by Rottweiler et al. (2023) as well. Pattern 2, ‘Avoidance and Distraction’, closely resembled Rottweiler et al.'s (2023) ‘Avoidance’ pattern. Rottweiler et al. (2023) also identified two patterns characterized by social support, our corresponding pattern was additionally characterized by expression (pattern 5 ‘Social Support and Expression’). Pattern 4, ‘Suppression’, did not occur in Rottweiler et al. (2023). This shows that the situational emotion regulation patterns of teachers are similar to those of students, but that they also employ an additional pattern characterized by an avoidance‐based strategy.

The five patterns suggest that certain strategies may enable or constrain the use of others. For example, the findings hint that expression and suppression might be mutually exclusive: these strategies are either employed in opposition to one another (patterns 1 ‘Average ER’, 3 ‘Low ER’, 4 ‘Suppression’ and 5 ‘Social Support and Expression’) or both are used only minimally (pattern 2 ‘Avoidance and Distraction’). Additionally, avoidance‐based strategies often co‐occur (patterns 2 ‘Avoidance and Distraction’ and 4 ‘Suppression’). Social support and expression seem to appear together, as seen in pattern 5, ‘Social Support and Expression’. It is possible that expression becomes viable only in social contexts where one can share emotions. In this scenario, the act of expressing emotion may not be the central issue; rather, it is the fact that a social partner can perceive the emotion, providing support either through reappraisal or by alleviating fears through shared rumination. Although speculative, it is plausible that rumination in a social context facilitates subsequent reappraisal, whereas isolated rumination may be less beneficial, potentially trapping individuals in negative thought cycles. Thus, rumination, when paired with social support, might serve a helpful function.

### Person‐level profiles

At the person‐level, four profiles of emotion regulation strategies with similar frequencies (ranging from 17.4% to 31.8%) emerged. While profile 4, ‘Uniform Distribution of ER Patterns’, was characterized by the use of multiple different situational patterns, all three other profiles were characterized by the dominant use of one or two situational patterns. Moreover, teachers in two profiles (1 ‘Predominantly Avoidance & Distraction’ and 2 ‘Predominantly Average ER’) relied on the use of one situational regulation pattern, while in one profile (3 ‘Predominantly Average ER and Low ER’), they predominantly used two situational patterns. This shows that some teachers (Profile 4%, 17.4%, *N* = 29) used different combinations of strategies flexibly in different situations, but the majority of teachers regulated their emotions predominantly with an invariable pattern of emotion regulation strategies (Profiles 1, 2 and 3, 82.6% in total). This finding is in line with Rottweiler et al. (2023), who found that only 31% of their investigated students resorted to the use of several situational emotion regulation patterns, whereas the other students relied on the dominant use of one situational pattern. Our findings may indicate that some teachers are not able to flexibly switch between emotion regulation patterns in different situations and instead rely on one or two single patterns. This might suggest a ‘stable’ use of specific strategies, with a certain degree of flexibility in adjusting these strategies to fit the specific situation. The entropy indicates that the probability that teachers will be assigned to the correct profile was .73, thus below the often‐considered acceptable threshold of .80. As the study was not designed for diagnostic reasons, the low value itself might not be as problematic. However, the result itself might hint at the interpretation that emotion regulation is not a very stable person‐specific behaviour and should rather be discussed at a situational level.

The profiles 1, ‘Predominantly Avoidance and Distraction’, and 4, ‘Uniform Distribution of ER Patterns’, were similar to the profiles from Rottweiler et al. (2023), namely ‘Predominantly Avoidance’ and ‘Multi ER Profiles’. Our profile 3 was characterized by the patterns ‘Average ER’ and ‘Low ER’, while the third profile from Rottweiler et al. (2023) was characterized only by ‘Predominantly Low ER’. In addition, we found another profile characterized by ‘Predominantly Average ER’ (profile 2). Overall, our findings, based on teachers, align with those of Rottweiler et al. (2023) regarding students, identifying similar emotion regulation profiles while also revealing an additional fourth profile at the person‐level. Based on theoretical considerations, it could be advantageous if teachers belonged to the flexible profile 4.

### Covariates

Our findings emphasize the emotion specificity of emotion regulation patterns (e.g., Harley et al., 2019; Rottweiler et al., 2023), as experiencing joy was relevant for the pattern probability. The results suggest that in negative, non‐teaching situations with average anger, an experience of joy above average is connected to an increased likelihood of using pattern 2, ‘Avoidance and Distraction’, and pattern 4, ‘Suppression’. This might indicate that teachers who rely on avoidance‐based rather than approach‐based strategies in negatively valenced situations may enhance their enjoyment by avoiding the situation, distracting themselves from unpleasant thoughts or suppressing other, more negative emotions in that situation. Vice versa, it might also indicate that teachers perceive these situations as sufficiently enjoyable that they could still regulate with avoidance, distraction or suppression as rather simpler, more superficial strategies. A possible fictitious example would be listening to music (strategy: avoidance) instead of doing organizational tasks, which takes you far enough away from the low‐valued situation to experience joy. It is very important to note here once again that our data are correlational in nature, and we cannot draw any conclusions about causal effects. Thus, it might be that a specific regulation behaviour elicits the emotion joy or that the experience of a specific level of joy elicits a specific regulation behaviour.

The covariate analyses were conducted in one combined model, as joy and anger are interrelated. We also calculated the models separately for both emotions, and the pairwise comparisons with Benjamini and Hochberg (1995) correction showed that anger had a significant impact when it was the only emotion covariate. This could be an indication of a complex interaction between the two emotions in the combined model.

In positive as well as in teaching situations, the probability of using patterns 2 ‘Avoidance and Distraction’ and 4 ‘Suppression’ declines, as there might be no need or no opportunity to use patterns that are characterized by avoidance‐based strategies. This finding aligns with the ERAS model (Harley et al., 2019), emphasizing the importance of the situation and the combined occurrence of strategies. Differentiating between low‐ and high‐evaluative situations may influence which patterns of emotion regulation strategies are applicable. In certain situations, such as teaching, some strategies like avoidance (e.g., leaving the situation) are not feasible.

The findings highlight the different roles of various emotion regulation patterns, especially pattern 2, ‘Distraction and Avoidance’, and pattern 4, ‘Suppression’, in positive and negative situations. In negative situations, individuals who use the ‘Distraction and Avoidance’ and ‘Suppression’ patterns reported experiencing relatively more joy than they typically would. However, in positive situations, where joy is naturally more intense, relying on distraction, avoidance and suppression may be unnecessary, leading to a lower likelihood of using patterns 2 and 4 compared to the ‘Average ER’ pattern. This suggests that the effectiveness of different emotion regulation patterns depends on the emotional context, with patterns 2 and 4 being more relevant in negative situations but less so in positive ones. Future studies could investigate differences in emotion regulation patterns in terms of low‐ vs. high‐evaluative and individual vs. social situations (Harley et al., 2019).

While experiencing positive teaching situations, teachers tend to regulate more frequently with a pattern characterized by suppression (pattern 4 ‘Suppression’). In these situations, teachers may choose to suppress their emotional experiences, as other strategies might be less feasible and less appropriate in these situations.

Our findings confirm theoretical assumptions (Gross, 1998b; Harley et al., 2019) and previous findings on the situation‐specificity of emotion regulation strategies (e.g., Rottweiler et al., 2023) and expand on previous research by investigating how daily situations are associated with specific emotion regulation combinations among teachers.

While individual strategies, namely avoidance, distraction and rumination, were associated with emotional exhaustion at the between‐level (see Table 1), no significant differences were found in the probability of emotional exhaustion and the combined use of strategies, that is, the profiles. This builds on previous findings that have examined associations between single emotion regulation strategies and well‐being (e.g., Chang, 2020; Doyle et al., 2024; Yin et al., 2016). The results suggest that none of our identified emotion regulation profiles, characterized by the predominant use of certain combinations of situation‐specific emotion regulation strategies, suffered from particularly elevated or dampened levels of emotional exhaustion. However, it is also possible that the method of measuring emotional exhaustion as a day‐specific variable, in contrast to the measures of the other study variables, has an effect, and future studies could attempt to assess both situation‐specific well‐being and person‐specific well‐being indicators as well as well‐being measures beyond emotional exhaustion.

### Implications

Although many teachers might not be as flexible in their use of different emotion regulation patterns, as demonstrated by the pattern‐specific profiles, the study still demonstrated, in line with previous studies, that the use of patterns of multiple emotion regulation strategies is related to the experienced emotion, the valence, and the context of the situation. Thus, our findings have implications for the design of emotion regulation training programs for teachers. Future training programs might consider the emotion‐ and situation‐specific nature of emotion regulation. It might be beneficial to educate teachers about theoretical models (Gross, 1998b; Harley et al., 2019) that highlight the importance of emotions and context in emotion regulation. Additionally, teachers might be introduced to a range of emotion regulation strategies (e.g., Smrtnik Vitulić & Prosen, 2022). Special emphasis should be placed on understanding how emotions and regulation strategies can vary depending on the situation. Participants should therefore have opportunities to experiment with different regulation strategies in various scenarios, such as through role‐plays that allow teachers to practice and explore diverse methods of emotion regulation. Finally, this approach could be incorporated into university‐based teacher training programs to better prepare student teachers.

### Limitations and further directions

Firstly, although the eight strategies selected cover all five emotion regulation families of Gross's process model (Gross, 1998b), it is conceivable that teachers use additional strategies to regulate their emotions. Future studies should also consider these other strategies. Secondly, the study focused on the investigation of two emotions, namely joy and anger, only. As teachers experience a variety of emotions during their everyday work life, it would be important to further investigate additional emotions in future studies. Thirdly, although diary studies enable realistic measurements while minimizing retrospective distortion (Almeida, 2005; Bolger et al., 2003), the survey conducted in the present study at the end of each day does not eliminate the possibility that teachers may not have been able to accurately report their strategies without bias. Future studies could aim to further reduce recall bias, for example, by using the experience sampling method (e.g., Rottweiler et al., 2023) to capture teachers' emotions and regulation strategies directly in the situation. Lastly, the present study, despite its extensive repeated‐measures design, rests on correlational data patterns and thus does not permit causal interpretation. Therefore, further research is necessary to untangle the associations between emotion regulation patterns and the emotions experienced. This could be implemented through dynamic network modelling (e.g., Daumiller et al., 2024 on students). Overall, our findings are preliminary and exploratory, warranting replication. If replicated, it would be valuable to explore whether teachers who demonstrate greater flexibility in using various emotion regulation strategies are also more adaptive in their overall emotion regulation.

## CONCLUSION

The present study contributes to the theoretical understanding of teachers' emotion regulation strategies and the patterns and profiles in which they occur. The results highlight that different emotion regulation patterns are both emotion‐ and situation‐specific, meaning that teachers regulate their emotions differently in varying situations. Based on our data, we cannot draw any conclusions about the adaptivity or non‐adaptivity of emotion regulation behaviour, nor do we attempt to do so. Instead, we raise the fundamental question of whether adaptive or maladaptive strategies exist at all in a universal sense. We propose that research should focus more on the combined use of different strategies depending on the actual emotional experience and the specific situation. Future training programs on emotion regulation for teachers should consider this emotion‐ and situation‐specific nature of emotion regulation.

## AUTHOR CONTRIBUTIONS

**Tanja Bross:** Conceptualization; formal analysis; investigation; methodology; project administration; writing – original draft; writing – review and editing. **Anne Christiane Frenzel:** Supervision; writing – review and editing. **Ulrike Elisabeth Nett:** Conceptualization; formal analysis; funding acquisition; investigation; methodology; project administration; supervision; resources; writing – review and editing.

## FUNDING INFORMATION

This work was funded by the Federal Ministry of Education and Research Germany as part of the joint ‘Teacher Training Quality Campaign’ of the federal and state governments (Grant number 01JA1809).

## CONFLICT OF INTEREST STATEMENT

The authors have no relevant financial or non‐financial interests to disclose.

## Data Availability

The data that support the findings of this study are available from the corresponding author upon reasonable request.

## Footnotes

^1^
According to Gross (1998b), rumination is one of the emotion regulation strategies within the family of attentional deployment that is sometimes considered maladaptive.

^2^
In Germany, the vocational track represents the basic track of the German three‐track secondary school system.

## References

[bjep12765-bib-0001] Aldao, A. (2013). The future of emotion regulation research: Capturing context. Perspectives on Psychological Science, 8(2), 155–172. 10.1177/1745691612459518 26172497

[bjep12765-bib-0002] Almeida, D. M. (2005). Resilience and vulnerability to daily stressors assessed via diary methods. Current Directions in Psychological Science, 14(2), 64–68. 10.1111/j.0963-7214.2005.00336.x

[bjep12765-bib-0003] Becker, E. S. , Keller, M. M. , Goetz, T. , Frenzel, A. C. , & Taxer, J. L. (2015). Antecedents of teachers' emotions in the classroom: An intraindividual approach. Frontiers in Psychology, 6, 635. 10.3389/fpsyg.2015.00635 26042067 PMC4436560

[bjep12765-bib-0004] Benjamini, Y. , & Hochberg, Y. (1995). Controlling the false discovery rate: A practical and powerful approach to multiple testing. Journal of the Royal Statistical Society. Series B, Methodological, 57, 289–300.

[bjep12765-bib-0005] Bolger, N. , Davis, A. , & Rafaeli, E. (2003). Diary methods: Capturing life as it is lived. Annual Review of Psychology, 54, 579–616. 10.1146/annurev.psych.54.101601.145030 12499517

[bjep12765-bib-0006] Bozdogan, H. (1987). Model selection and Akaike's information criterion (AIC): The general theory and its analytical extensions. Psychometrika, 52(3), 345–370. 10.1007/BF02294361

[bjep12765-bib-0007] Braun, S. S. , Schonert‐Reichl, K. A. , & Roeser, R. W. (2020). Effects of teachers' emotion regulation, burnout, and life satisfaction on student well‐being. Journal of Applied Developmental Psychology, 69, 101151. 10.1016/j.appdev.2020.101151

[bjep12765-bib-0008] Burić, I. , & Frenzel, A. C. (2019). Teacher anger: New empirical insights using a multi‐method approach. Teaching and Teacher Education, 86, 102895. 10.1016/j.tate.2019.102895

[bjep12765-bib-0009] Burić, I. , & Frenzel, A. C. (2020). Teacher emotional labour, instructional strategies, and students' academic engagement: A multilevel analysis. Teachers and Teaching, 27(5), 335–352. 10.1080/13540602.2020.1740194

[bjep12765-bib-0010] Burić, I. , Slišković, A. , & Macuka, I. (2017). A mixed‐method approach to the assessment of teachers' emotions: Development and validation of the teacher emotion questionnaire. Educational Psychology, 38(3), 325–349. 10.1080/01443410.2017.1382682

[bjep12765-bib-0011] Butler, R. (2007). Teachers' achievement goal orientations and associations with teachers' help seeking: Examination of a novel approach to teacher motivation. Journal of Educational Psychology, 99(2), 241–252. 10.1037/0022-0663.99.2.241

[bjep12765-bib-0012] Carver, C. S. , Scheier, M. F. , & Weintraub, J. K. (1989). Assessing coping strategies: A theoretically based approach. Journal of Personality and Social Psychology, 56(2), 267–283. 10.1037//0022-3514.56.2.267 2926629

[bjep12765-bib-0013] Chang, M.‐L. (2020). Emotion display rules, emotion regulation, and teacher burnout. Frontiers in Education, 5, 90. 10.3389/feduc.2020.00090

[bjep12765-bib-0014] Chang, M.‐L. , & Taxer, J. (2020). Teacher emotion regulation strategies in response to classroom misbehavior. Teachers and Teaching, 27(5), 353–369. 10.1080/13540602.2020.1740198

[bjep12765-bib-0015] Daumiller, M. , Nett, U. , & Putwain, D. (2024). Complex dynamics: Investigation of within‐ and between‐person relationships between achievement emotions and emotion regulation during exam preparation through dynamic network modeling. Journal of Educational Psychology, 116(7), 1267–1282. 10.1037/edu0000883

[bjep12765-bib-0016] Doyle, N. B. , Downer, J. T. , & Rimm‐Kaufman, S. E. (2024). Understanding Teachers' emotion regulation strategies and related teacher and classroom factors. School Mental Health, 16(1), 123–136. 10.1007/s12310-023-09624-8

[bjep12765-bib-0017] Fombouchet, Y. , Pineau, S. , Perchec, C. , Lucenet, J. , & Lannegrand, L. (2023). The development of emotion regulation in adolescence: What do we know and where to go next? Social Development, 32(4), 1227–1242. 10.1111/sode.12684

[bjep12765-bib-0018] Ford, B. Q. , Gross, J. J. , & Gruber, J. (2019). Broadening our field of view: The role of emotion Polyregulation. Emotion Review, 11(3), 197–208. 10.1177/1754073919850314

[bjep12765-bib-0019] Frenzel, A. C. (2014). Teacher emotions. In R. Pekrun & L. Linnenbrink‐Garcia (Eds.), International handbook of emotions in education (1st ed., pp. 494–519). Routledge.

[bjep12765-bib-0020] Frenzel, A. C. , Daniels, L. , & Burić, I. (2021). Teacher emotions in the classroom and their implications for students. Educational Psychologist, 56(4), 250–264. 10.1080/00461520.2021.1985501

[bjep12765-bib-0021] Frenzel, A. C. , & Goetz, T. (2007). Emotionales Erleben von Lehrkräften beim Unterrichten. Zeitschrift für Pädagogische Psychologie, 21(3–4), 283–295.

[bjep12765-bib-0022] Garnefski, N. , & Kraaij, V. (2007). The cognitive emotion regulation questionnaire. European Journal of Psychological Assessment, 23(3), 141–149. 10.1027/1015-5759.23.3.141

[bjep12765-bib-0023] Goetz, T. , Bieg, M. , Ludtke, O. , Pekrun, R. , & Hall, N. C. (2013). Do girls really experience more anxiety in mathematics? Psychological Science, 24(10), 2079–2087. 10.1177/0956797613486989 23985576

[bjep12765-bib-0024] Grommisch, G. , Koval, P. , Hinton, J. D. X. , Gleeson, J. , Hollenstein, T. , Kuppens, P. , & Lischetzke, T. (2020). Modeling individual differences in emotion regulation repertoire in daily life with multilevel latent profile analysis. Emotion, 20(8), 1462–1474. 10.1037/emo0000669 31478725

[bjep12765-bib-0025] Gross, J. J. (1998a). Antecedent‐ and response‐focused emotion regulation: Divergent consequences for experience, expression, and physiology. Journal of Personality and Social Psychology, 74(1), 224–237. 10.1037/0022-3514.74.1.224 9457784

[bjep12765-bib-0026] Gross, J. J. (1998b). The emerging field of emotion regulation: An integrative review. Review of General Psychology, 2(3), 271–299.

[bjep12765-bib-0027] Gross, J. J. (2015). Emotion regulation: Current status and future prospects. Psychological Inquiry, 26(1), 1–26. 10.1080/1047840x.2014.940781

[bjep12765-bib-0028] Gross, J. J. , & John, O. P. (2003). Individual differences in two emotion regulation processes: Implications for affect, relationships, and well‐being. Journal of Personality and Social Psychology, 85(2), 348–362. 10.1037/0022-3514.85.2.348 12916575

[bjep12765-bib-0029] Gwet, K. L. (2008). Computing inter‐rater reliability and its variance in the presence of high agreement. British Journal of Mathematical and Statistical Psychology, 61(1), 29–48. 10.1348/000711006X126600 18482474

[bjep12765-bib-0030] Harley, J. M. , Pekrun, R. , Taxer, J. L. , & Gross, J. J. (2019). Emotion regulation in achievement situations: An integrated model. Educational Psychologist, 54(2), 106–126. 10.1080/00461520.2019.1587297

[bjep12765-bib-0031] Heiy, J. E. , & Cheavens, J. S. (2014). Back to basics: A naturalistic assessment of the experience and regulation of emotion. Emotion, 14(5), 878–891. 10.1037/a0037231 24999913

[bjep12765-bib-0032] Hipp, J. R. , & Bauer, D. J. (2006). Local solutions in the estimation of growth mixture models. Psychological Methods, 11(1), 36–53. 10.1037/1082-989X.11.1.36 16594766

[bjep12765-bib-0033] Jeon, L. , & Ardeleanu, K. (2020). Work climate in early care and education and Teachers' stress: Indirect associations through emotion regulation. Early Education and Development, 31(7), 1031–1051. 10.1080/10409289.2020.1776809

[bjep12765-bib-0034] Keller, M. M. , Chang, M. L. , Becker, E. S. , Goetz, T. , & Frenzel, A. C. (2014). Teachers' emotional experiences and exhaustion as predictors of emotional labor in the classroom: An experience sampling study. Frontiers in Psychology, 5, 1442. 10.3389/fpsyg.2014.01442 25566124 PMC4263074

[bjep12765-bib-0035] Koole, S. L. (2009). The psychology of emotion regulation: An integrative review. Cognition and Emotion, 23(1), 4–41. 10.1080/02699930802619031

[bjep12765-bib-0036] Li, L. , Huang, L. , & Liu, X. (2023). Primary school teacher's emotion regulation: Impact on occupational well‐being, job burnout, and resilience. Psychology in the Schools, 60(10), 4089–4101. 10.1002/pits.22982

[bjep12765-bib-0037] Lo, Y. , Mendell, N. R. , & Rubin, D. B. (2001). Testing the number of components in a normal mixture. Biometrika, 88(3), 767–778. 10.1093/biomet/88.3.767

[bjep12765-bib-0038] Lukočienė, O. , Varriale, R. , & Vermunt, J. K. (2010). The simultaneous decision(s) about the number of lower‐ and higher‐level classes in multilevel latent class analysis. Sociological Methodology, 40(1), 247–283. 10.1111/j.1467-9531.2010.01231.x

[bjep12765-bib-0039] Maslach, C. , Jackson, S. E. , & Leiter, M. P. (1996). Maslach burnout inventory manual (3rd ed.). Consulting Psychologists Press.

[bjep12765-bib-0040] Meihami, H. , & Esmaili, S. (2024). TAFL and TEFL Teachers' emotional vulnerability and emotion regulation strategies in online classes. The Asia‐Pacific Education Researcher, 33, 913–929. 10.1007/s40299-023-00804-3

[bjep12765-bib-0041] Messineo, L. , & Tosto, C. (2022). Perceived stress and affective experience in Italian teachers during the COVID‐19 pandemic: Correlation with coping and emotion regulation strategies. European Journal of Psychology of Education, 38(3), 1271–1293. 10.1007/s10212-022-00661-6 PMC9734932 40479454

[bjep12765-bib-0042] Parkinson, B. , & Totterdell, P. (1999). Classifying affect‐regulation strategies. Cognition & Emotion, 13(3), 277–303. 10.1080/026999399379285

[bjep12765-bib-0043] Pekrun, R. (2006). The control‐value theory of achievement emotions: Assumptions, corollaries, and implications for educational research and practice. Educational Psychology Review, 18(4), 315–341. 10.1007/s10648-006-9029-9

[bjep12765-bib-0044] Pekrun, R. , Goetz, T. , Titz, W. , & Perry, R. P. (2002). Academic emotions in Students' self‐regulated learning and achievement: A program of qualitative and quantitative research. Educational Psychologist, 37(2), 91–106. 10.1207/S15326985EP3702_4

[bjep12765-bib-0045] Rottweiler, A.‐L. , Stockinger, K. , & Nett, U. E. (2023). Students’ regulation of anxiety and hope—A multilevel latent profile analysis. Emotion, 23(7), 1891–1903. 10.1037/emo0001200 36595386

[bjep12765-bib-0046] Rottweiler, A.‐L. , Taxer, J. L. , & Nett, U. E. (2018). Context matters in the effectiveness of emotion regulation strategies. AERA Open, 4(2), 2332858418778849. 10.1177/2332858418778849

[bjep12765-bib-0047] Schmidt, J. , Klusmann, U. , Lüdtke, O. , Möller, J. , & Kunter, M. (2017). What makes good and bad days for beginning teachers? A diary study on daily uplifts and hassles. Contemporary Educational Psychology, 48, 85–97. 10.1016/j.cedpsych.2016.09.004

[bjep12765-bib-0048] Schwarz, G. (1978). Estimating the dimension of a model. The Annals of Statistics, 6(2), 461–464.

[bjep12765-bib-0049] Smrtnik Vitulić, H. , & Prosen, S. (2022). Teachers' experience and regulation of anger and fear in the school context. Educational Psychology, 42(6), 714–729. 10.1080/01443410.2021.2024514

[bjep12765-bib-0050] Sutton, R. E. (2004). Emotional regulation goals and strategies of teachers. Social Psychology of Education, 7, 379–398.

[bjep12765-bib-0051] Sutton, R. E. , & Knight, C. C. (2006). Teachers' emotion regulation. In A. V. Mitel (Ed.), Trends in Educational Psychology (pp. 107–136). Nova Publish.

[bjep12765-bib-0052] Taxer, J. L. , & Gross, J. J. (2018). Emotion regulation in teachers: The “why” and “how”. Teaching and Teacher Education, 74, 180–189. 10.1016/j.tate.2018.05.008

[bjep12765-bib-0053] Troy, A. S. , Shallcross, A. J. , & Mauss, I. B. (2013). A person‐by‐situation approach to emotion regulation: Cognitive reappraisal can either help or hurt, depending on the context. Psychological Science, 24(12), 2505–2514. 10.1177/0956797613496434 24145331

[bjep12765-bib-0054] Vermunt, J. K. , & Magidson, J. (2021). Latent GOLD 6.0. Statistical Innovations Inc.

[bjep12765-bib-0055] Vuong, Q. H. (1989). Likelihood ratio tests for model selection and non‐nested hypotheses. The Econometric Society, 57(2), 307–333.

[bjep12765-bib-0056] Wang, H. , Burić, I. , Chang, M.‐L. , & Gross, J. J. (2023). Teachers' emotion regulation and related environmental, personal, instructional, and well‐being factors: A meta‐analysis. Social Psychology of Education, 26(6), 1651–1696. 10.1007/s11218-023-09810-1

[bjep12765-bib-0057] Yin, H. , & Guo, Y. (2024). A person‐centered analysis of Hong Kong kindergarten teachers' emotion regulation: Profiles, characteristics and relations. European Journal of Education, 59, e12687. 10.1111/ejed.12687

[bjep12765-bib-0058] Yin, H. , Huang, S. , & Wang, W. (2016). Work environment characteristics and teacher well‐being: The mediation of emotion regulation strategies. International Journal of Environmental Research and Public Health, 13(9), 907. 10.3390/ijerph13090907 27649216 PMC5036740

